# Dominance of *Dibothriocephalus ditremus* over *Dibothriocephalus dendriticus* (Cestoda: Diphyllobothriidea) in salmonids from Lake Kalandsvatn (Western Norway) revealed by molecular genotyping

**DOI:** 10.1016/j.crpvbd.2026.100394

**Published:** 2026-06-02

**Authors:** Ivica Králová-Hromadová, Eva Čisovská Bazsalovicsová, Alžbeta Radačovská, Lucia Dinisová, Egil Karlsbakk

**Affiliations:** a Institute of Parasitology, Slovak Academy of Sciences, Hlinkova 3, Košice, 04001, Slovakia; b The University of Veterinary Medicine and Pharmacy in Košice, Komenského 73, Košice, 04001, Slovakia; c Department of Biological Sciences, University of Bergen, P.O. Box 7803, Bergen, N-5020, Norway

**Keywords:** Diphyllobothriid tapeworms, *Salmo trutta*, *Salvelinus alpinus*, Molecular genotyping, Relative abundance, Prevalence, Intensity of infection

## Abstract

The occurrence of *Dibothriocephalus dendriticus* and *Dibothriocephalus ditremus* in salmonids (brown trout *Salmo trutta* and Arctic charr *Salvelinus alpinus*) from Lake Kalandsvatn in Western Norway was studied. Plerocercoids were identified by external morphology and PCR-based analysis using Dd_8 microsatellite primers. Of 1313 plerocercoids, 121 (9.2%) were identified as *D. dendriticus* and 1192 (90.8%) as *D. ditremus*. The study provides the first record of *D. ditremus* in Lake Kalandsvatn. The prevalence of *D. dendriticus* was 51.3% in trout and 76.9% in charr, while *D. ditremus* prevalence was higher (76.9% in trout and 100% in charr). The intensities of *D. dendriticus* infection were also lower (trout 1–17; charr 1–20) compared to *D. ditremus* (trout 1–221; charr 2–86). The higher relative abundance of *D. ditremus* compared to *D. dendriticus* was detected in most trout and charr. The dominance of *D. ditremus* probably reflects the feeding strategies and the higher abundance of its definitive hosts, piscivorous birds.

## Highlights

• *Dibothriocephalus dendriticus* and *Dibothriocephalus ditremus* were studied in Lake Kalandsvatn, Norway.
• Plerocercoids were identified by PCR-based analysis using microsatellite Dd_8 primers.
• Of 1313 plerocercoids, 121 (9.2%) were identified as *D. dendriticus* and 1192 (90.8%) as *D. ditremus.*
• Intensity of infection was 1–17 (trout) and 1–20 (charr) (*D. dendriticus*) and 1–221 (trout) and 2–86 (charr) (*D. ditremus*).
• The higher relative abundance and dominance of *D. ditremus* reflects feeding strategies of its piscivorous bird hosts.

## Graphical abstract

**Figure:**
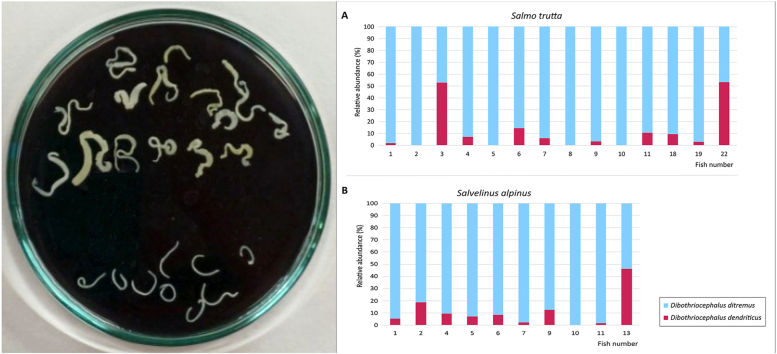

## 1 Introduction

The life cycles of diphyllobothriid tapeworms *Dibothriocephalus dendriticus* (Nitzsch, 1824) (syn. *Diphyllobothrium dendriticum*) and *Dibothriocephalus ditremus* (Creplin, 1825) (syn. *Diphyllobothrium ditremum*) are confined to freshwater environments; their development requires two intermediate hosts (freshwater copepods and fishes), aquatic birds (definitive hosts of both species), and piscivorous mammals (definitive hosts only for *D. dendriticus*). The most frequent and common fish hosts of both tapeworms are salmonids, such as the Atlantic salmon *Salmo salar*, brown trout *Salmo trutta*, Arctic charr *Salvelinus alpinus*, vendace (European cisco) *Coregonus albula,* common whitefish *Coregonus lavaretus*, and European grayling *Thymallus thymallus.* In addition to salmonids, the European smelt *Osmerus eperlanus* (Osmeridae), three-spined stickleback *Gasterosteus aculeatus* and ninespine stickleback *Pungitius* *pungitius* (Gasterosteidae), and burbot *Lota* *lota* (Lotidae) also serve as hosts of *D. dendriticus* and *D. ditremus* (for a review, see Supplementary Table 1 in Králová-Hromadová et al., 2025c).

Plerocercoids of *D. dendriticus* and *D. ditremus* are present in capsules and often co-occur in the same fish host; they can be morphologically identified and distinguished from each other immediately after dissection based on their external morphology, mainly size and shape (Andersen et al., 1987). However, these parameters may vary according to different media and fixative solutions (e.g. ethanol, formol saline, physiological saline) and also according to the fish host, with the most profound differences between the infections in three-spined stickleback and brown trout (Halvorsen, 1970; Andersen et al., 1987). Besides the morphological artefacts caused by different fixation or freezing procedures, differentiation between larvae may also be challenging due to their morphological plasticity and high intraspecific variability. As a result, plerocercoids were identified as *Diphyllobothrium/Dibothriocephalus* spp. in several studies in Iceland (Kristmundsson and Richter, 2009), Norway (Moccetti et al., 2019; Paterson et al., 2019; Olk et al., 2020) and Scotland (UK) (Hooker et al., 2016; Rochat et al., 2022). Problematic identification of hardly distinguishable larvae can be resolved by molecular taxonomy using a properly chosen DNA marker. Recently, genotyping based on the microsatellite Dd_8 primers was designed for differentiation of plerocercoids of *D. dendriticus* and *D. ditremus* (Králová-Hromadová et al., 2025b). This approach has so far been applied only in the identification of *Dibothriocephalus* spp. from salmonids in Iceland (Králová-Hromadová et al., 2025c). Accurate identification of larvae is important not only for exact data on taxonomy and biodiversity, but also for interpretation of biological data, e.g. the dominance of *D. ditremus* over *D. dendriticus* in the same fish host, which has been documented in Norway, Sweden, and Iceland (Halvorsen, 1970; Halvorsen and Wissler, 1973; Henricson, 1977; Králová-Hromadová et al., 2025c).

The aim of this study was to evaluate the intensity of infection, prevalence, and relative abundance of *D. dendriticus* and *D. ditremus* in salmonids from Lake Kalandsvatn in Western Norway, a region with scarce and more than 55-years old data on *Diphyllobothrium/Dibothriocephalus* spp. infections (Vik, 1959; Mørch, 1968; Halvorsen, 1970). The main focus was to determine which species of *Dibothriocephalus* dominates in salmonids in the studied lake*.* The identification of *D. dendriticus* and *D. ditremus* in individual fish hosts was based initially on external morphology followed by genotyping using Dd_8 species-specific microsatellite primers, which were applied for the Norwegian population for the first time. The future use of the sample-set from Kalandsvatn in population genetics conducted on a global scale is also discussed.

## 2 Materials and methods

### 2.1 Parasite material

Plerocercoids were isolated from 52 fish (39 brown trout *S. trutta* and 13 Arctic charr *S. alpinus*) from Lake Kalandsvatn (60.27403°N, 5.39483°E), the largest lake (surface area 3.5 km^2^) in Bergen Municipality, Vestland County, Western Norway. Fish were caught and killed by local members of a group of landowners managing the lake (Landowners Association). Plerocercoids were isolated from capsules localized in the abdominal cavity and internal organs of infected fish and rinsed in physiological saline solution. Initial identification of the larvae was based on their external morphology according to Andersen et al. (1987). All plerocercoids were preserved in 96% ethanol for further molecular identification.

### 2.2 Molecular genotyping

Genomic DNA from each larva was isolated using the QIAamp® DNA Mini Kit (Qiagen, Hilden, Germany) according to the manufacturer’s recommendations. The DNA was stored in deionised water at −20 °C. Species-specific PCR-based genotyping of all plerocercoids of *Dibothriocephalus* spp. was performed with the forward primer Dd_8_F (5′-CGT CTA TGA TCA CGC ATG TCA-3′) and the reverse primer Dd_8_R (5′-CGC TGT AGG ATT AGA TTC ACA CG-3′), which amplify the Dd_8 microsatellite locus in *D. dendriticus.* PCR was performed in a total volume of 20 μl with 10–20 ng of genomic DNA, 10 pmol of each of the two primers and 1× PCR Master Mix (Thermo Fisher Scientific Inc., Waltham, USA). The PCR amplification conditions were 5 min at 95 °C as an initial denaturation step, followed by 40 cycles of 30 s at 95 °C, 1 min at 60 °C, 1 min at 72 °C, and a final polymerization step of 10 min at 72 °C. The PCR products (∼250 bp), which were amplified only in *D. dendriticus* DNA, were visualised on a 1.5% agarose gel.

### 2.3 Statistical analyses

Prevalence values with 95% Wilson Score confidence interval (CI) were calculated using a web application (https://www.statskingdom.com/).

## 3 Results

A total of 1313 plerocercoids were isolated from infected salmonids from Kalandsvatn. The initial identification was based on external morphology, focusing on body size and structure. Plerocercoids of *D. dendriticus* were larger (> 20 mm), segmented, and more mobile, while those of *D. ditremus* were smaller (< 20 mm), had a smooth appearance with few body folds, and were more rigid in their movements. In total, 1192 (90.8%) plerocercoids were morphologically identified and genotyped as *D. ditremus* and 121 (9.2%) larvae were genotyped as *D. dendriticus*. There was a slight discrepancy between the initial morphological identification of *D. dendriticus* and the results of genotyping, because 13 small-sized larvae with not very evident segmentation were first misidentified as *D. ditremus*, but later on genotyped as *D. dendriticus*. The results showed that even though the morphological identification of live larvae kept in physiological saline is highly reliable, a multidisciplinary approach can make the results of identification even more accurate. Of note, the identity of all *D. dendriticus* and > 200 *D. ditremus* larvae has been confirmed by the sequences of 891 bp mitochondrial *cox*1 (unpublished data from ongoing research).

**Fig. 1:**
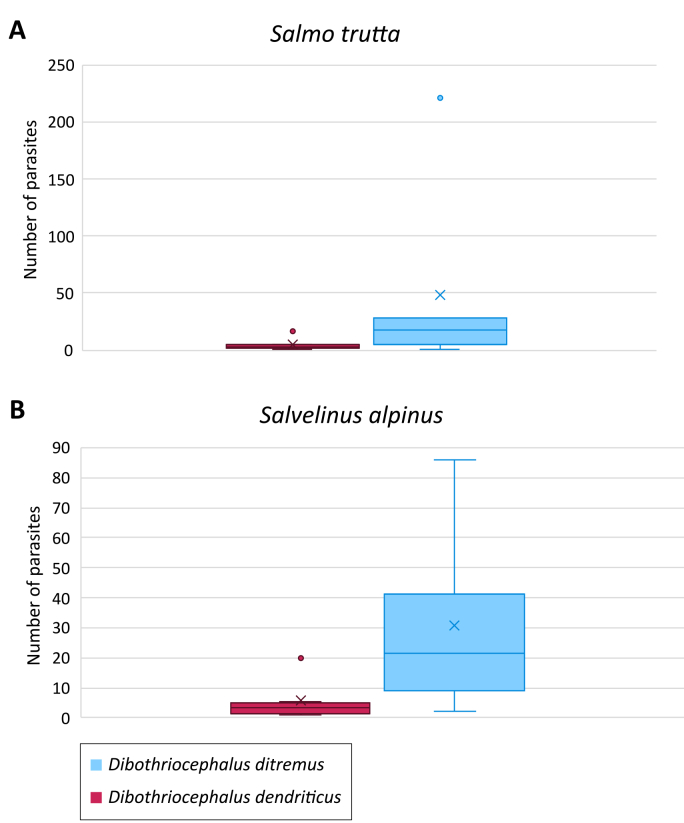
Box-and-whisker plots showing the intensity of infection with *Dibothriocephalus dendriticus* and *Dibothriocephalus ditremus* in salmonids from Lake Kalandsvatn. **A** Brown trout *Salmo trutta*. **B** Arctic charr *Salvelinus alpinus*. *Notes*: Lines inside the boxes indicate the median, and the symbol “×” indicates the mean; circles represent the maximum number of parasites.

The prevalence of *D. dendriticus* was 51.3% in trout and 76.9% in charr. A higher prevalence was detected for *D. ditremus*, 76.9% in trout and 100% in charr (Table 1). Of the 39 examined trout, 32 (82%) were infected with at least one tapeworm species, while seven trout were negative for *Dibothriocephalus* spp. infection (Table 2). All 13 charr were infected with at least one of the tapeworm species (Table 3). The intensity of infection with *D. dendriticus* was lower in both fish species (trout 1–17; charr 1–20) compared to substantially higher values recorded for *D. ditremus* (trout 1–221; charr 2–86) (Table 1). The graphical representation of the mean, maximum, and minimum intensity of infection is shown in Fig. 1.

**Table 1:** Summary data on prevalence and intensity of infection with *Dibothriocephalus dendriticus* and *Dibothriocephalus ditremus* in the brown trout *Salmo trutta* and Arctic charr *Salvelinus alpinus* from Lake Kalandsvatn, Western Norway.

Fish species (sample size)	*N*	*Dibothriocephalus dendriticus*					*Dibothriocephalus ditremus*				
		*n*	P (95% CI) (%)	*N*	Range I	Mean I	*n*	P (95% CI) (%)	*N*	Range I	Mean I
*Salmo trutta* (*n* = 39)	912	20	51.3 (36.2–66.2)	76	1–17	3.8	30	76.9 (61.6–87.3)	836	1–221	27.9
*Salvelinus alpinus* (*n* = 13)	401	10	76.9 (49.7–91.8)	45	1–20	4.5	13	100 (−)	356	2–86	27.4
Total (*n* = 52)	1313			121					1192		

*Abbreviations: N*, number of larvae; *n*, number of infected fish; P, prevalence; I, intensity of infection, 95% CI, Wilson score confidence interval.

**Table 2:** Summary data on *Dibothriocephalus dendriticus* and *Dibothriocephalus ditremus* infections in the brown trout *Salmo trutta* from Lake Kalandsvatn, Western Norway.

Fish number	Length (mm)	Sex	*N*	No. of *D. dendriticus* (%)	No. of *D. ditremus* (%)
1	250	M	52	1 (1.9)	51 (98.1)
2	256	F	30	0 (0)	30 (100)
3	251	F	17	9 (52.9)^b^	8 (47.1)
4	263	F	238	17 (7.1)	221 (92.9)
5	269	M	53	0 (0)	53 (100)
6	258	F	14	2 (14.3)	12 (85.7)
7	305	F	169	10 (5.9)	159 (94.1)
8	253	F	24	0 (0)	24 (100)
9	273	M	29	1 (3.4)	28 (96.6)
10	321	F	25	0 (0)	25 (100)
11	216	M	66	7 (10.6)	59 (89.4)
12	262	F	6^a^	0 (0)	6 (100)
13	197	F	3^a^	0 (0)	3 (100)
14	256	F	5^a^	2 (40)	3 (60)
15	161	F	4^a^	0 (0)	4 (100)
16	216	M	1^a^	0 (0)	1 (100)^c^
17	264	F	9^a^	1 (11.1)	8 (88.9)
18	247	F	21	2 (9.5)	19 (90.5)
19	212	F	65	2 (3.1)	63 (96.9)
20	259	M	–	–	–
21	247	M	10^a^	1 (10)	9 (90)
22	239	M	15	8 (53.3)^b^	7 (46.7)
23	202	M	5^a^	0 (0)	5 (100)
24	204	M	5^a^	0 (0)	5 (100)
25	277	M	4^a^	0 (0)	4 (100)
26	246	M	7^a^	2 (28.6)	5 (71.4)
27	223	M	3^a^	0 (0)	3 (100)
28	244	M	1^a^	1 (100)^c^	0 (0)
29	222	M	–	–	–
30	235	F	1^a^	1 (100)^c^	0 (0)
31	214	M	7^a^	1 (14.3)	6 (85.7)
32	261	M	–	–	–
33	291	F	10^a^	3 (30)	7 (70)
34	245	F	–	–	–
35	254	F	9^a^	2 (22.2)	7 (77.8)
36	242	F	–	–	–
37	276	M	–	–	–
38	279	M	–	–	–
39	213	F	4^a^	3 (75)	1 (25)
Total			912	76 (8.3)	836 (91.7)

*Notes:* %, relative abundance (percent of the total number of *Dibothriocephalus* spp. plerocercoids in individual fish); *N*, total no. of larvae; –, uninfected fish.

a Relative abundance calculated for fish with ≤10 *Dibothriocephalus* spp. plerocercoids should be interpreted with caution.

b Fish with >10 *Dibothriocephalus* spp. plerocercoids with a slightly higher relative abundance of *D. dendriticus* than *D. ditremus*.

c Relative abundance in fish infected with one larva.

**Table 3:** Summary data on *Dibothriocephalus dendriticus* and *Dibothriocephalus ditremus* infections in the Arctic charr *Salvelinus alpinus* from Lake Kalandsvatn, Western Norway.

Fish number	Length (mm)	Sex	*N*	No. of *D. dendriticus* (%)	No. of *D. ditremus* (%)
1	269	F	38	2 (5.3)	36 (94.7)
2	271	F	106	20 (18.9)	86 (81.1)
3	267	F	2^a^	0 (0)	2 (100)
4	254	M	53	5 (9.4)	48 (90.6)
5	249	M	14	1 (7.1)	13 (92.9)
6	251	M	24	2 (8.3)	22 (91.7)
7	238	F	45	1 (2.2)	44 (97.8)
8	234	M	8^a^	5 (62.5)	3 (37.5)
9	217	M	16	2 (12.5)	14 (87.5)
10	268	F	16	0 (0)	16 (100)
11	252	F	60	1 (1.7)	59 (98.3)
12	281	F	6^a^	0 (0)	6 (100)
13	260	F	13	6 (46.2)^b^	7 (53.8)
Total			401	45 (11.2)	356 (88.8)

*Notes:* %, relative abundance (percent of the total number of *Dibothriocephalus* spp. plerocercoids in individual fish); *N*, total no. of larvae.

a Relative abundance calculated for fish with ≤ 10 *Dibothriocephalus* spp. plerocercoids should be interpreted with caution.

b Fish with > 10 *Dibothriocephalus* spp. plerocercoids with a high relative abundance of *D. dendriticus*.

The body length of the examined charr ranged within a narrow range of 217 to 281 mm (Table 3), while the length of trout was between 161 and 321 mm (Table 2). Even though the smallest trout (161 and 197 mm) were infected with a low number of larvae (4 and 3, respectively), all negative trout were larger than 200 mm, and the biggest trout (321 mm) was infected only with 25 plerocercoids (Table 2).

**Fig. 2:**
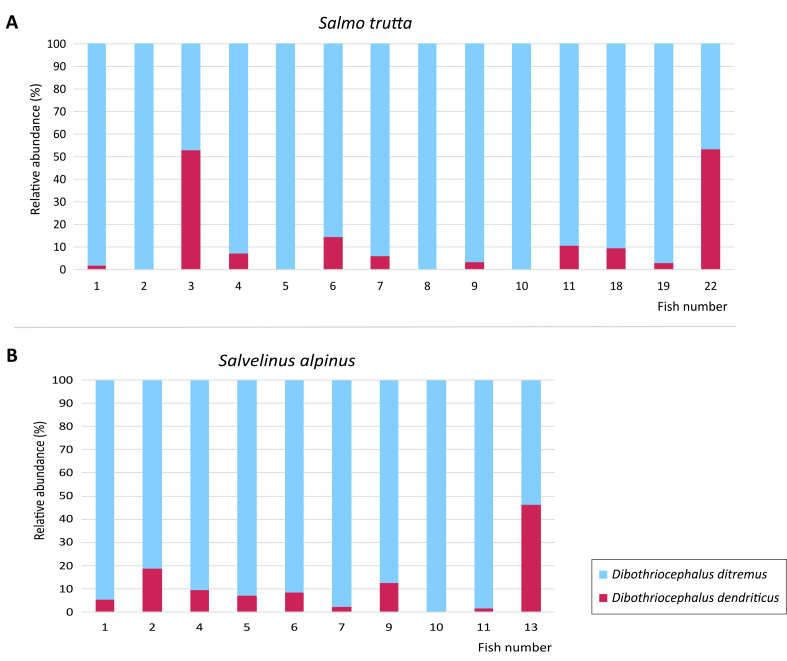
Relative abundance of *Dibothriocephalus dendriticus* and *Dibothriocephalus ditremus* in individual fish hosts. **A** Brown trout *Salmo trutta*. **B** Arctic charr *Salvelinus alpinus*. Details on the fish are provided in Table 2, Table 3.

The relative abundance of *D. dendriticus* and *D. ditremus* plerocercoids in individual fish hosts was calculated for all infected fish (Table 2, Table 3). Of 32 infected trout, 18 fish harboured ≤ 10 plerocercoids (Table 2). The calculation of relative abundance in those individuals must be interpreted with caution, especially for fish in which one larva was found (trout nos. 16, 28, and 30) (Table 2). In the remaining 14 trout, the total number of plerocercoids ranged from 14 to 238, allowing a more reliable calculation of relative abundance. In 12 trout, substantially lower values were determined for *D. dendriticus* (1.9–14.3%), while *D. ditremus* was present at a markedly higher ratio (85.7–100%) (Table 2). The graphical interpretation of the relative abundance of *D. dendriticus* and *D. ditremus* in individual trout with >10 plerocercoids is presented in Fig. 2A, highlighting distinct dominance of *D. ditremus* in all but two fish (nos. 3 and 22) (Table 2).

Regarding the 13 infected Arctic charr, three of them harboured ≤ 10 plerocercoids (Table 3). In the remaining 10 charr, the total number of plerocercoids ranged from 13 to 106. In nine charr, substantially lower values of relative abundance were determined for *D. dendriticus* (1.7–18.9%), than for *D. ditremus* (81.1–100%), while in one fish (no. 13) the relative abundance of *D. dendriticus* was higher (46.2%) (Table 3). The graphical interpretation of the relative abundance in individual charr with >10 plerocercoids is presented in Fig. 2B, showing the dominance of *D. ditremus* in the substantial majority of fish.

## 4 Discussion

The present study provided new data on *Dibothriocephalus* spp. infections in salmonids from Lake Kalandsvatn in Western Norway, a region for which very limited information has been published more than five decades ago (Vik, 1959; Mørch, 1968; Halvorsen, 1970). The only data on *Dibothriocephalus* spp. in fish from Kalandsvatn were published by Mørch (1968), who observed plerocercoids of *D. dendriticus* in both charr and trout, but found *D. ditremus* to be absent in the lake. The present findings indicate significant changes in the abundance of *D. ditremus* in Lake Kalandsvatn over the past decades, which can be explained by an increased occurrence of bird definitive hosts, including the great cormorant *Phalacrocorax carbo*. This bird is a confirmed definitive host of *D. ditremus* (Bylund, 1971; Kanarek and Zaleśny, 2014) and has become abundant in Lake Kalandsvatn in the last decades (E. Karlsbakk, personal observation).

Diphyllobothriid tapeworms can persist in the fish host for several years and the larvae accumulate within the fish due to repeated infections, hence the intensity of infection tends to increase with the increasing age/length of host (Henricson, 1977). The present data were not entirely consistent with this general theory, most probably due to the narrow length range of the fish examined.

A substantially higher intensity of infection was detected for *D. ditremus* compared to *D. dendriticus* in majority of fish hosts from Kalandsvatn. Dominance of *D. ditremus* over *D. dendriticus* had previously been observed in Norway (Halvorsen, 1970; Halvorsen and Wissler, 1973), Sweden (Henricson, 1977), and Iceland (Králová-Hromadová et al., 2025c). Halvorsen (1970) explained this phenomenon by a higher tolerance of eggs, procercoids and plerocercoids of *D. ditremus* to lower temperatures as well as by the feeding strategy of aquatic birds. *Dibothriocephalus dendriticus* parasitises gulls, namely *Larus canus* (Vik, 1957; Bakke, 1985), which can catch smaller and less infected fish, such as three-spined sticklebacks (Halvorsen, 1970). In contrast, divers (*Gavia* spp.), goosanders and mergansers, which are hosts of *D. ditremus* (Vik, 1957; Bylund, 1971; Ryzhikov et al., 1985), prey on larger and more heavily infected trout, thus shedding more *D. ditremus* eggs into the lake.

The relationship between parasite burden and the feeding strategy of fish, namely copepod transmission route in charr, piscivorous transmission route in trout, and predation of larger trout on small charr, were studied in detail in fish from Takvatn (Henriksen et al., 2019). In agreement with the present findings, a lower prevalence of *D. dendriticus* compared to *D. ditremus* was documented in both trout and charr (Henriksen et al., 2019). However, in contrast to our data, Henriksen et al. (2019) detected a higher prevalence of *D. dendriticus* in trout than in charr, which was related to the size and feeding strategy of trout. Larger trout (> 350 mm in length) included in their diet charr, which were more heavily infected with *D. dendriticus* than fishes of smaller size (e.g. sticklebacks) (Kuhn et al., 2015). This was certainly not the case in Kalandsvatn, because trout of larger size (> 350 mm in length), in which an effect of predation would be documented, were not examined in the present study (Table 2).

## 5 Conclusions

*Dibothriocephalus dendriticus* and *D. ditremus* are interesting parasite models for studies on their phylogeographical origin and intercontinental dispersal, as their definitive hosts are highly mobile aquatic birds undertaking long-distance migrations. The first prerequisite for robust genetic analyses is a broad collection of reliably identified populations from distinct geographic localities. While genetic diversity and intercontinental dispersal were assessed for subarctic and temperate populations of *D. dendriticus* (Králová-Hromadová et al., 2025a), a comprehensive study on the interrelationships, origin, and migratory routes of European populations of *D. ditremus* has been conducted only on populations from Iceland (Dinisová et al., 2025). Molecularly identified *D. ditremus* individuals from Kalandsvatn in Western Norway provide a large sample-set that will be incorporated into future population genetic studies of this tapeworm on a global scale.

## Ethical approval

Not applicable. All fish analysed in the present study were caught and killed by the Landowners Association managing the Lake Kalandsvatn and then provided to the authors, who performed an autopsy and isolated the larvae.

## CRediT authorship contribution statement

**Ivica Králová-Hromadová:** Conceptualization, Funding acquisition, Investigation, Methodology, Project administration, Supervision, Writing - original draft, Writing - review & editing. **Eva Čisovská Bazsalovicsová:** Investigation, Methodology, Writing - review & editing. **Alžbeta Radačovská:** Investigation, Methodology, Writing - review & editing. **Lucia Dinisová:** Visualisation, Writing - review & editing. **Egil Karlsbakk:** Resources, Writing - review & editing.

## Funding

This work was financially supported by the 10.13039/501100005357Slovak Research and Development Agency (project no. APVV-23-0390).

## Declaration of competing interests

The authors declare that they have no known competing financial interests or personal relationships that could have appeared to influence the work reported in this paper.

## Data Availability

The data supporting the conclusions of this article are included within the article.
